# Dynamic evolution of anodic biofilm when maturing under different external resistive loads in microbial fuel cells. Electrochemical perspective

**DOI:** 10.1016/j.jpowsour.2018.08.031

**Published:** 2018-10-01

**Authors:** Grzegorz Pasternak, John Greenman, Ioannis Ieropoulos

**Affiliations:** a Bristol BioEnergy Centre, Bristol Robotics Laboratory, Coldharbour Lane, BS16 1QY Bristol, UK; b Faculty of Chemistry, Wrocław University of Science and Technology, Wyb. Wyspiańskiego 27, 50-370, Wrocław, Poland

**Keywords:** External resistance, Internal resistance, Dynamics, Evolution, Continuous flow, Structure

## Abstract

Appropriate inoculation and maturation may be crucial for shortening the startup time and maximising power output of Microbial Fuel Cells (MFCs), whilst ensuring stable operation. In this study we explore the relationship between electrochemical parameters of MFCs matured under different external resistance (R_ext_) values (50 Ω - 10 kΩ) using non-synthetic fuel (human urine). Maturing the biofilm under the lower selected R_ext_ results in improved power performance and lowest internal resistance (R_int_), whereas using higher R_ext_ results in increased ohmic losses and inferior performance. When the optimal load is applied to the MFCs following maturity, dependence of microbial activity on original R_ext_ values does not change, suggesting an irreversible effect on the biofilm, within the timeframe of the reported experiments. Biofilm microarchitecture is affected by R_ext_ and plays an important role in MFC efficiency. Presence of water channels, EPS and precipitated salts is distinctive for higher R_ext_ and open circuit MFCs. Correlation analysis reveals that the biofilm changes most dynamically in the first 5 weeks of operation and that fixed R_ext_ lefts an electrochemical effect on biofilm performance. Therefore, the initial conditions of the biofilm development can affect its long-term structure, properties and activity.

## Highlights

• Dynamic changes of biofilm matured under different external loads are investigated.
• Most dynamic changes are observed in the first 3 weeks of operation.
• External resistance lower than internal resistance improves maturing of the biofilm.
• Internal resistance is dependent on external resistance applied in maturing phase.
• External resistance irreversibly affects the biofilm properties and performance.

## Graphical abstract

**Figure:**
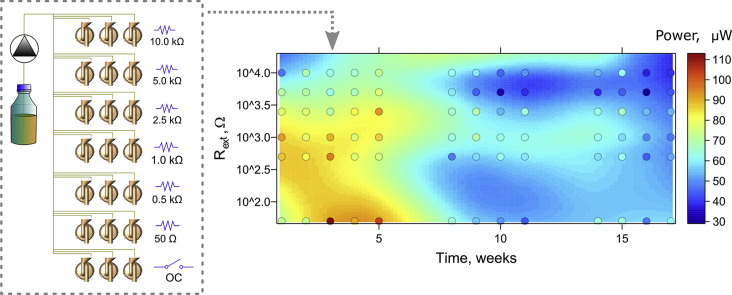

## 1 Introduction

Microbial Fuel Cell (MFC) technology uses electroactive bacteria to produce electricity through oxidation of organic matter. The technology has received increased attention over past decades [1]. The bioelectrochemical reactions take place in anodic and cathodic components of the MFC have found many potential applications in the fields of wastewater treatment, electricity generation, biogas production, biosensors and bioelectrochemical synthesis [[2], [3], [4], [5], [6], [7], [8], [9], [10]]. Among various carbon sources that have been demonstrated as a fuel in MFCs human urine has proved to be a good substrate due to its high conductivity [11]. The ongoing development of the MFCs focuses on developing electrode materials, catalysts, membranes separating anodic and cathodic chambers, design and scale-up of the MFC-based systems [2,[12], [13], [14], [15], [16], [17]].

Despite the broad interest in many engineering aspects of the MFCs, the most crucial role is played by the electroactive bacteria, which form the biofilm on the electrode's surface and generate electrical power from their population-level metabolism. The biofilm is a complex matrix of microorganisms and extracellular compounds which is considered to be very stable, albeit possessing physiological adaptive mechanisms [18] many of which are expressed during the initial biofilm formation period.

Several research groups have previously reported on power performance and start up times when the electroactive community has been matured under different poised anode potentials. For example the anodic biofilm formed by *Geobacter sulfurreducens* gives highest power performance and lowest MFC start-up time, when matured under a potential range between 0 and 400 mV vs SHE (standard hydrogen electrode). This optimal potential range promoted the biofilm growth and corresponding power density of the MFCs [19]. The study reported by Aelterman et al. showed, that optimal biofilm growth and activity was obtained when the anode was poised at −200 mV vs Ag/AgCl electrode, although the original source of the bacterial inoculum was not mentioned [20]. More recently, Zhu et al. reported, that acclimating the biofilm with positive potentials may lead to the decay of the power overshoot phenomenon which leads to improved power performance [21].

Easier ways of controlling the potential of MFC electrodes is by applying an external load (R_ext_), which does not require any specialist equipment that could be limiting in particular for field applications. Comprehensive investigation on the effect of R_ext_ on biofilm formation and activity has been reported by Zhang et al. [22]. The authors investigated the ohmic range of 10–1000 Ω and indicated that optimal R_ext_ for their MFC setup was found to be 50Ω, although biofilm matured under 10 Ω produced the highest current. The study also showed that R_ext_ had an impact on the presence of extracellular polymeric substances (EPS) of the biofilm, and a more recent study showed that EPS plays a role in biofilm performance and in turn, power generation [23]. The influence of three different R_ext_ values on biofilm activity (after the maturing phase) was also studied by Jung and Regan [24]. The authors focused on methane production and the inhibition of methanogenesis was found to occur in parallel with the highest power efficiency for MFCs fed with acetate and operating under lowest (150 Ω) R_ext_. Earlier studies also demonstrated the relationship of R_ext_ applied during operation (after maturing phase) with performance of the MFCs in relation to the fuel supply and the best results were obtained when MFCs were operated under R_ext_ closer to internal resistance (R_int_) [25]. External resistance was also found to be a factor influencing diversity of the bacterial community [24,26,27].

Although significant work has been done to understand the interactions of R_ext_ with the biofilm, very limited knowledge is available on dynamic evolution of biofilm subjected to various external loads. Since the biofilm forms both stable and adaptive structure, such knowledge is indispensable to develop appropriate strategies for inoculation and operation of MFCs. It is therefore important to determine, whether the conditions applied to the biofilm in the initial stage of development may leave a structural and electrochemical profile and irreversibly affect its performance thereafter.

The aim of this study was to determine the temporal and long-term effects of fixed and dynamically-changed external resistance on changes of biofilm parameters and resulting MFC performance in time. The results revealed the irreversible effects that the initial R_ext_ causes to the biofilm, which may subsequently either induce or inhibit the power performance of the MFCs in long-term perspective. This is the first of two papers in series, where we have focused on analysis of electrochemical parameters. The second part of this study will focus on biological parameters of the biofilm.

## 2 Experimental

### 2.1 MFC construction and operation

**Fig. 1:**
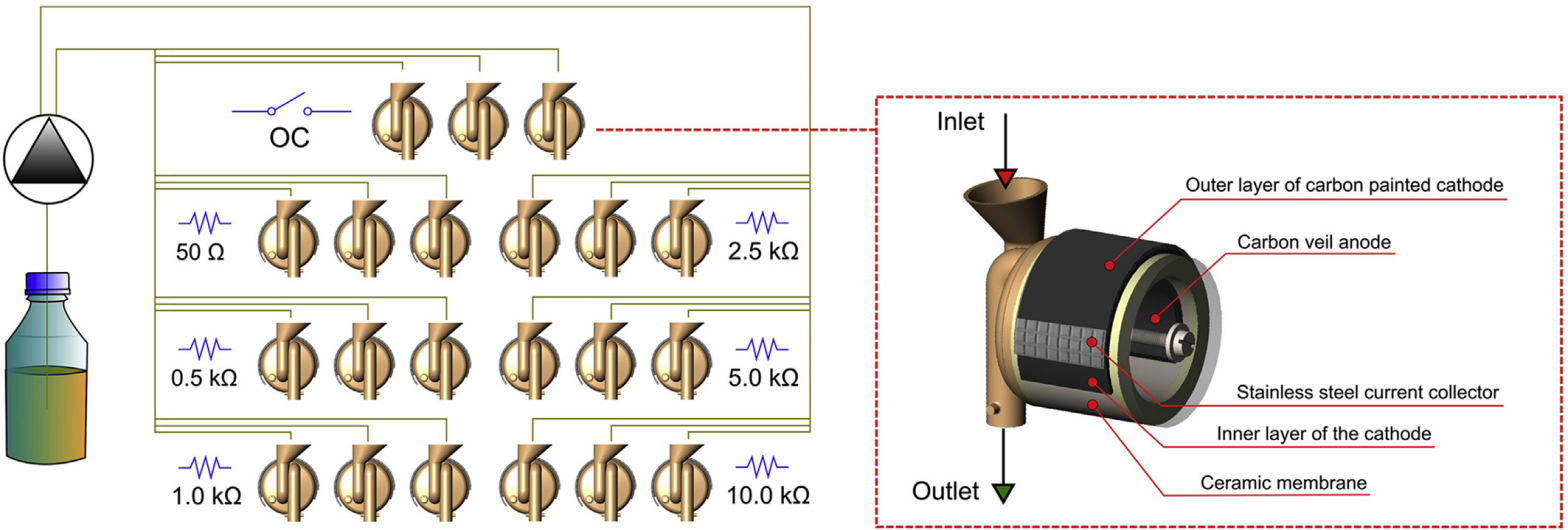
Schematic representation of experimental design and MFC design. The experimental setup consisted of predefined external loads only in the first stage (weeks 1–5) of the experiment. In the second stage (weeks 6–17) external load was changed dynamically to the optimal value determined for each individual MFC. Individual graphical elements of this image were derived from our previous works [30,31].

The single chamber MFCs were built as described in detail in our previous work [28]. In brief, earthenware ceramic material was used both as the separator and housing for the anodic chamber (Fig. 1). The external side of the ceramic cylinder was supplied with carbon-painted cathode (carbon loading of 14.08 mgC cm^−2^) and stainless steel wire mesh, acting as a current collector. The volume of an empty MFC anodic chamber was 11.4 mL. Carbon fiber veil (20 g m^−2^, PRF Composite Materials, Dorset, UK) was used as the anodes with total surface area of 252 cm^2^. A 3D-printed Nanocure^®^ RCP30-resin lid with inlet and outlet tubes was used as a front panel of the vertically positioned MFCs.

All of the MFCs were manufactured manually in the same manner and used to build an array of 21 electrically and fluidically isolated MFCs. The array consisted of 7 sets of triplicate MFCs. Each triplicate was operating under different electrochemical conditions, controlled by the external load, connected to each individual MFC.

In the first stage of experiments, the external resistance (R_ext_) was predefined for each triplicate, as shown on Fig. 1. The array consisted of MFCs connected to 50, 500, 1000, 5000 and 10000 Ω, as well as the open circuit (OC) control. The electroactive biofilm was grown at the anodes using these predesigned R_ext_ values for approximately 6 weeks. Activated sludge derived from the aerobic chamber of municipal wastewater treatment plant (Wessex Water, Saltford, UK) was used to inoculate the MFCs. After 24 h, activated sludge was removed from the MFCs and replaced with the fresh human urine as a fuel. The MFCs were operated in continuous flow conditions. The fuel was delivered to the MFC-array using multichannel peristaltic pump (Watson Marlow, USA) at a constant flow rate of 0.12 L d^−1^. Between stages 1 and 2, the MFCs were fed with gradually increasing flow rates to determine the impact on power performance. Before the second stage, the flow rate was brought back to a constant value of 0.12 L d^−1^.

The second stage of the experiment took place between 7 and 17 weeks of operation and was conducted in the same hydraulic conditions. In the second stage, the R_ext_ were adjusted dynamically, using optimal values determined individually for each MFCs, excluding OC control for which the open circuit conditions were maintained. Prior to the 17th week, the MFC cathodes were washed with deionised water due to the ongoing accumulation of inorganic salts at their surface, which deteriorated performance.

### 2.2 Dynamic electrochemical behaviour of the biofilm

Electrochemical behaviour of the biofilm was tracked over time through polarisation experiments and real time temporal power performance monitoring. Polarisation experiments were carried out each week. In the second stage, the optimal R_ext_ values determined from power curves were applied to each MFCs to simulate maximal power point tracking (MPPT) conditions. The polarisation experiments were conducted using automated variable resistor system, known as the resistorstat [29]. Each polarisation run consisted of 1 MΩ–3.75 Ω resistance range. Each value was connected to the MFC for a period of 5 min. The power performance of the MFCs was monitored with 34972 A Data Acquisition unit (Agilent Technologies, USA) with the data logging sample rate set to 3 min. The current was calculated according to Ohm's law: I = V/R, where V is the measured voltage in Volts (V) and R is the value of the external resistance. The power output P in Watts (W) was calculated using equation: P = I x V.

### 2.3 Environmental electron scanning microscopy

The biofilm structure was investigated by field emission environmental scanning microscopy, coupled with Energy-dispersive X-ray spectroscopy (Philips XL-30). Samples of the biofilm were collected from the anode at the end of the experimental period and fixed with 4% glutaraldehyde in 0.1 M PBS buffer. Subsequently, the samples were rinsed with water and air-dried for 12 h at room temperature.

### 2.4 Data processing and statistical analysis

Experimental data were first processed using Microsoft Excel 2010 and further analysed and visualized using R GUI package (v. 3.4.2). The polarisation experiments data was processed and visualized using SciDAVis (v. D001) software. Investigating the dynamic effects of R_ext_ on the biofilm properties and activity was conducted by local polynomial regression fitting, as well as by determining the Pearson's correlation coefficients between R_int_, R_ext_, OCV (open circuit voltage), power and current, extracted from polarisation experiments. The data was further visualized through the correlogram.

## 3 Results and discussion

All experiments were carried out using fresh human urine, collected on a daily basis as a fuel and delivered under constant flow rate. Therefore, the measured pH of fresh urine ranged from 6.15 to 6.29 and the average conductivity was equal to 11.76 ± 0.76 mS.

### 3.1 Electrochemical behaviour of biofilm

**Fig. 2:**
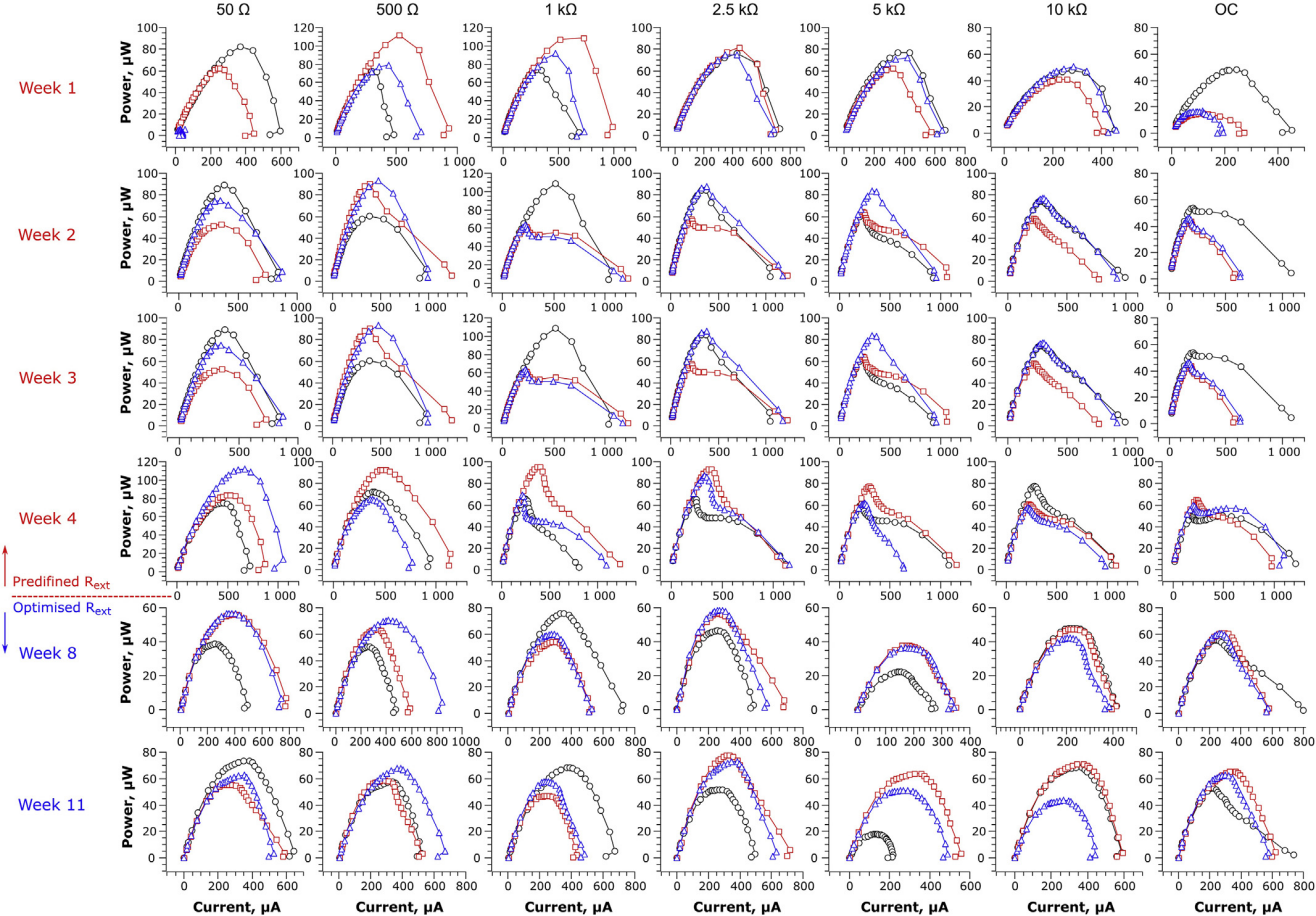
Electrochemical behaviour of MFCs represented by matrix of power curves, obtained from polarisation experiments during the first and second stages of the experiment. Each column in the matrix represents MFCs with predefined external resistance values, which are valid for the weeks 1–4. Starting from week 8, the R_ext_ values were continuously adjusted to the optimum (simulating MPPT). Each graph represents three individual MFC replicates.

It is known, that the shape of a polarisation and power curves and the corresponding losses are greatly dependent on the activity of electroactive bacteria [32,33]. Data derived from the polarisation experiments were presented in the form of power curves, as shown in Fig. 2. The results reported in this study comprised the highest coverage of R_ext_ within the operational range of MFCs when compared to previous studies available in the literature. Previous works used up to four R_ext_ values below 10 kΩ, where the highest divergence of the data may be expected [22,[24], [25], [26], [27]].

Maturing the MFCs under various loads resulted in achieving similar, symmetrical power curves across all the R_ext_ values. Nevertheless, the most uniform MFC characteristics between individual replicates were observed for 2.5 kΩ, resulting in the smallest variance of electrochemical parameters: OCV, I, P and R_int_. The highest dispersion of electrochemical parameters detected in polarisation experiments were reported for the two extreme R_ext_ values: 50 Ω and OC. The most significant concentration (mass transfer) losses were observed for 10 kΩ load, while the best performing individual MFCs were reported for 500 Ω and 1 kΩ. All of the MFCs revealed only negligible overshoot phenomenon, despite this being reported for early stage of biofilm development, as well as starvation and sub-optimal running conditions, and related to its metabolic rate and activity [21,34]. The lack of overshoot stays in line with our previous study, where the earthenware appeared to be the most favourable ceramic membrane material to create the appropriate MFC microenvironment which induces electroactive biofilm formation and activity [35].

In the second week of operation, significant changes in the MFC characteristics were observed. All of the MFCs operating under the load within the range 2.5 kΩ - OC, and 2 out of 3 MFCs operating under 1 kΩ, underperformed. Those MFCs showed significant, rapid drop of the performance in the central part of the power curves, corresponding to the ohmic losses. In contrast, the MFCs connected to R_ext_ of 50 and 500 Ω did not show such unfavourable changes. This trend continued until the end of the first stage of the experiment and became more significant each week, showing deterioration of the performance. Decrease of the power performance during polarisation was mainly caused by the drop of potential, while current values were similar or higher than those observed for MFCs operating under 50 and 500 Ω. The electrochemical data observed for MFCs operating under R_ext_≥1.0 kΩ and OC control, showed that ohmic losses were responsible for the gradual deterioration of power performance observed with time and with increasing R_ext_. Since the MFCs were operating in the same hydraulic conditions and were manufactured in the same way, the observed ohmic losses were probably caused by the formation of biofilm with suboptimal electrochemical properties, which may have been the result of both the R_ext_ and the low (but constant) flow rate. The MFCs were operating below the optimal value, which was found to be between 0.43 and 0.89 L d^−1^ (Figure S2). Suboptimal biofilm properties (physical, chemical and biological) could have resulted in achieving a highly resistive biofilm, which could negatively affect the overall internal resistance of the MFC. As reported by Nikhil et al., biofilm conductivity was the determining factor for the performance of MFCs inoculated with *Geobacter sulfurreducens* [36]. Such results are supported by the findings of McLean et al. who observed that 100 Ω - matured *Shewanella oneidensis* biofilm had lower thickness (<5 μm), than the biofilm matured under near-OC conditions (1.0 MΩ, >50 μm). Similar results were found by Read et al. during the initial stages of biofilm formation [37]. Reaching similar values of current, within the tested resistors, suggests that even suboptimally formed biofilm, contains sufficient numbers of electroactive bacteria, capable of delivering electrons to the electrode surface. This finding can be partially explained by the work of Jung et al. [24] who showed that dominating, electroactive species belonging to *Geobacteraceae* are present in the quantities of the same order of magnitude in biofilms operating under various R_ext_ values or even in OC conditions. Nevertheless, their study was constructed in a different way, i.e. the MFCs were initially matured for 3 weeks under the same R_ext_ after which various pre-defined loads were used (150–9800 Ω). More detailed ecological studies have reported significant effects of anodic potential levels on biofilm composition and consequently, performance of these bioelectrochemical systems, which may in turn play a key role in achieving desirable electrochemical properties [[38], [39], [40]].

The differences in biofilm properties were also revealed during the flow rate experiment (Figure S2). The results indicate, that only the 50 Ω-matured biofilm was capable of utilising nutrients at the highest flow/supply rate (0.888 L d^−1^). In parallel, the 50 Ω-biofilm was more susceptible to starvation (0.008 L d^−1^), which led to a drop of power output when compared to other R_ext_. These data perhaps suggest that the 50Ω biofilm had developed mechanisms for more efficient conversion of nutrients into electricity when the feeding rate is sufficiently high, but underdeveloped the ability to accumulate store energy reserves in such form as EPS (Fig. 5) which could be used when the feeding rate is low. Flow rate is an important parameter that is bound to affect the biofilm as a whole. Previous reports [41] describe how the flow rate affects the biofilm growth rate and in turn power output. However, the biofilm structure and how this may be affected by flow rate, is an area that requires dedicated investigation.

**Fig. 3:**
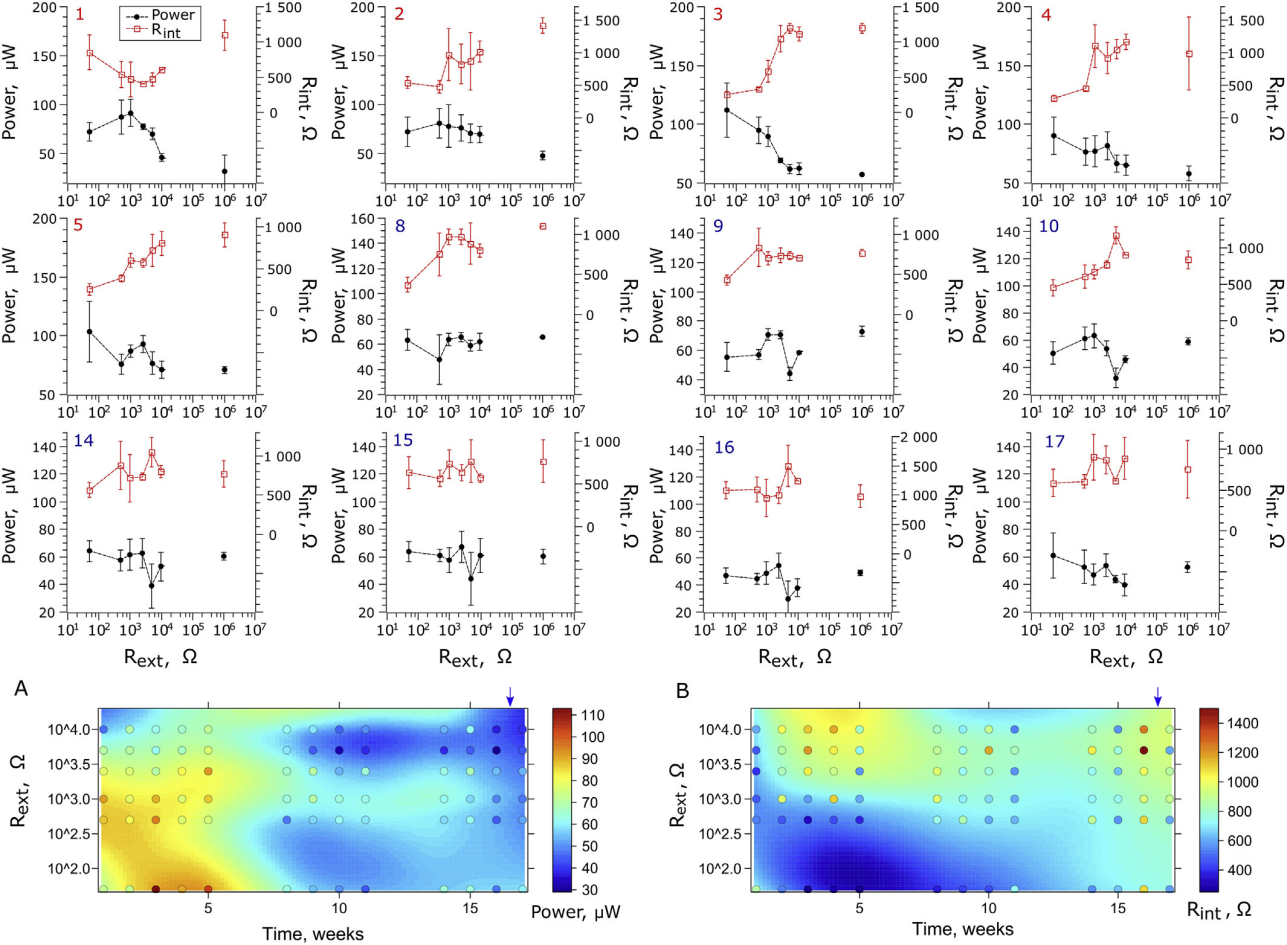
Evolution of the power performance and internal resistance of MFCs and biofilm matured under different external resistance values and open circuit (here designated as 10^6^ Ω) conditions. The numbers in the upper-left corner of each graph indicate the week of operation. The R_ext_ labels reflect the real, predefined external resistance for weeks 1–5 and original external resistance of the same MFC when the external resistance was changed to follow an MPPT procedure (weeks 8–17). The colour intensity of each point (plots A and B) refers to the measured value, while colour intensity of the surface refers to the regression model. The regression model included OC (10^6^ Ω) control for fitting, which was not shown in the plotting range for reasons of clarity. The points represent mean ± SD (plots 1–17) and mean values (plots A and B). Blue arrows indicate rinsing the cathodes with distilled water. (For interpretation of the references to colour in this figure legend, the reader is referred to the Web version of this article.)

**Fig. 4:**
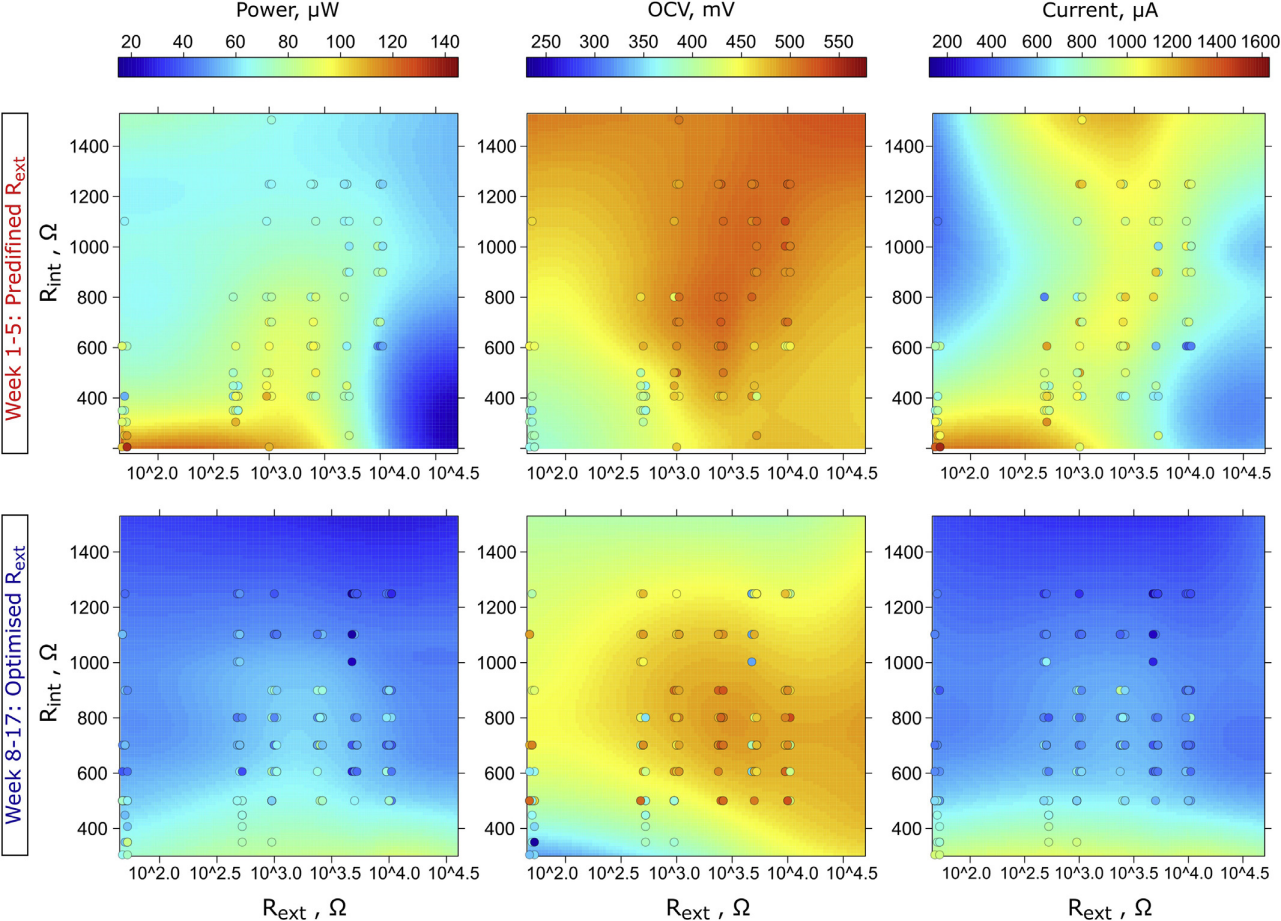
Simultaneous effect of external and internal resistance on electrochemical parameters of MFCs in two stages of experimental period. The labels on x-axes reflect the real, predefined external resistance for weeks 1–5 and original external resistance for the same MFCs once the simulated MPPT procedure was applied (weeks 8–17). The colour intensity of each point refers to the measured value, while colour intensity of the surface refer to the regression model. The regression model was also fitted using OC (10^6^ Ω) control, which was not included in the plotting range for reasons of clarity. (For interpretation of the references to colour in this figure legend, the reader is referred to the Web version of this article.)

**Fig. 5:**
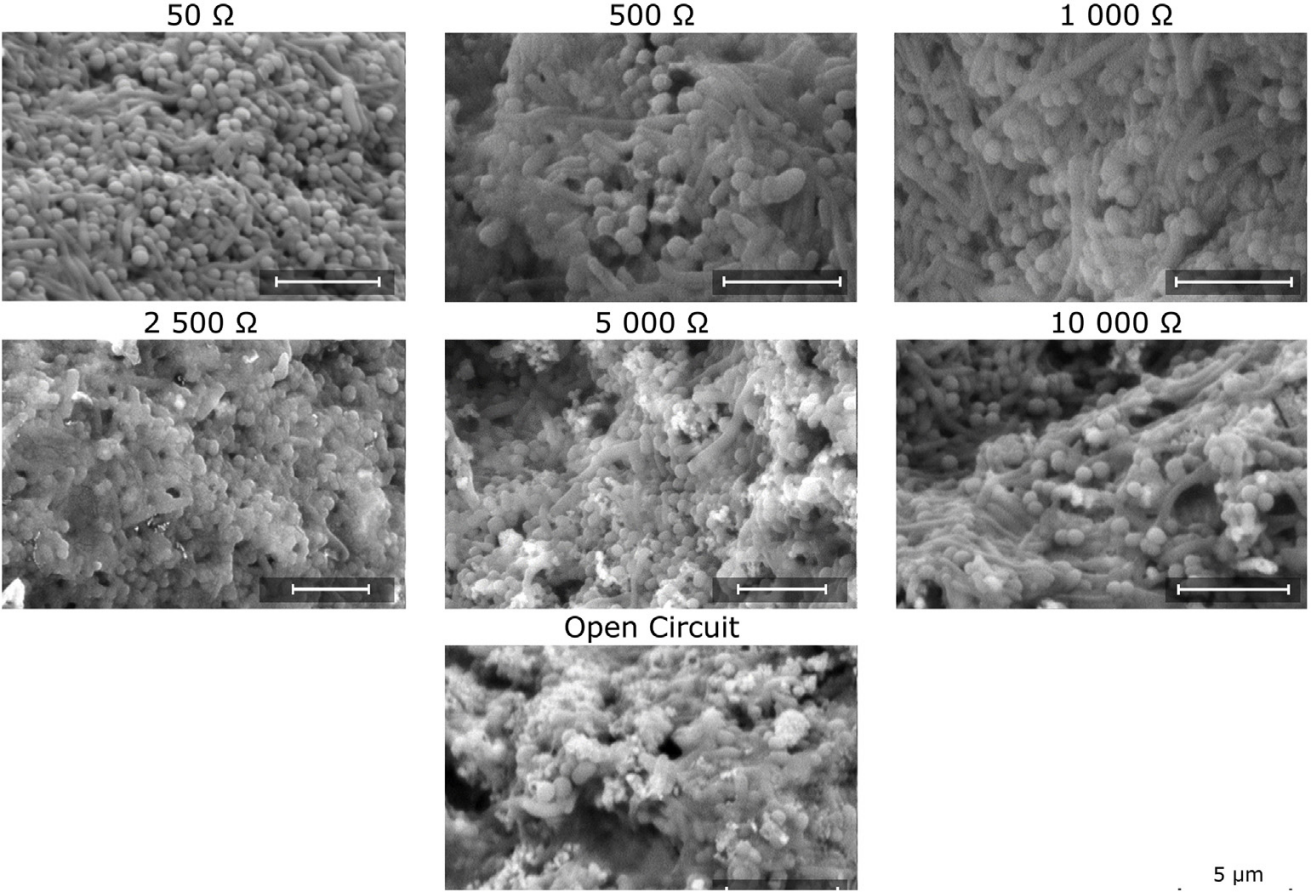
ESEM images showing changes in biofilm three-dimensional architecture, composition and occurrence of inorganic precipitates (bright spots), when matured under different external loads.

After 5 weeks of operation (stage 2), the pre-defined resistor values were replaced by the optimal R_ext_ values (derived from the polarisation data), adjusted to the optimum value at one-week intervals (simulating an MPPT approach). Changing the R_ext_ of the MFCs to follow their optimal values (when R_ext_ = R_int_ [33]) resulted in changes to the shape of MFC power curves. The ohmic losses previously observed in stage 1 were no longer visible. Such an interesting phenomenon suggests, that there was perhaps a change in biofilm behaviour that adjusted to the new electrochemical conditions. This could be explained by the increased oxidation of the carbon-energy substrate due to higher metabolic rates, since the new resistors connected to the MFCs had lower resistance, when compared to the stage 1. Higher metabolic rates are proportional to higher microbial growth rates and hence biomass density of the electroactive species. In a perfusable electrode system this maintains a fixed thickness biofilm, as the outer layers, which no longer have direct contact with the electrode, are constantly washed out. This results in a thinner biofilm and improves the diffusion of nutrients into the biofilm and conductivity (as indicated by R_int_ – Fig. 3). A thinner biofilm will also result in a significant reduction in extracellular polymeric substances (EPS) as previously reported by Zhang et al. [22]. The present study, suggests that R_int_ could dynamically change over time, as also alluded to by Winfield et al. [34].

### 3.2 Changes of power and internal resistance over time

To further investigate the impact of R_ext_ on biofilm maturing, the dynamic changes in R_int_ and power over time were also monitored (Fig. 3). In the first week of operation, the highest power performance was observed for 1 kΩ-matured MFCs and reached 91.6 ± 14.0 μW, whilst the R_int_ reached a value of 471.3 ± 247.5 Ω. The lowest R_int_ value was also observed for 1 kΩ and was equal to 205.4 Ω (derived from the polarisation experiment). Thus, in the first week, the use of 1 kΩ R_ext_ created the most favourable conditions to obtain the lowest R_int_.

During the first 3 weeks, the highest power performance was observed for 1 kΩ MFCs. In the following weeks the maximum power output was recorded for lower R_ext_ values: 500 Ω MFCs in week 2 (81.3 ± 15.0 μW) and 50 Ω MFCs in week 3 (112.2 ± 23.1 μW). Similarly, the minimum R_int_ values were observed for MFCs matured under R_ext_ = 500 Ω in week 2 (473.5 ± 93.8 Ω) and R_ext_ = 50 Ω in week 3 (253.8 ± 40.6 Ω). During the whole 1st stage of biofilm maturing, the lowest power performance and the highest R_int_ values were observed for the MFCs operating under higher R_ext_ values and under open circuit conditions, whilst the highest power values and lowest R_int_ values were recorded for the MFCs operating under lower R_ext_ values. The relationship between R_ext_, R_int_ and power was the most notable in the 3rd week of biofilm maturing, which appears to be the critical time required for the biofilm to develop. Furthermore, the highest performance of the MFCs during the entire experiment was also observed in the 3rd week of operation and was recorded for the MFCs matured under the lowest Ohmic value, 50 Ω (112.2 ± 23.2 μW). Following the 3rd week of operation, the increase of R_int_ with increasing R_ext_ was observed even in the 2nd stage of operation, where optimal R_ext_ values were applied following the simulated MPPT method. The internal resistance showed similar (but negative) trends that could be observed for power performance, which was reflected by similar patterns developed by the MFCs through time (Fig. 3A–B). The correlation between those two factors was later confirmed by statistical analysis. The internal resistance may be affected by various parameters such as hydraulic and environmental conditions [25,42,43]. In this study the hydraulic and other operational conditions remained constant, even though the flow rate was relatively low for this type of MFC. Therefore, such dynamic changes of internal resistance, in particular in the early period of operation (stage 1) resulted from ongoing biofilm development. Such changes may affect the conductivity of the biofilm, which is directly correlated with the current density by reducing the resistance of the electron flow and lowering the activation energy required for electron transfer between biofilm and the anode [36].

Interestingly, at the end of the 1st stage, a local power optimum was observed for 2.5 kΩ and became even more distinctive in the 5th week of operation. The 2.5 kΩ MFCs reached 93.0 ± 7.1 μW, which corresponds to 89.8% of the performance observed for 50 Ω MFCs. This state was also observed in the later stage of the biofilm growth, when the optimal R_ext_ resistors were applied to the MFCs (stage 2). Local polynomial regression fitting (Fig. 3A and 3. B) revealed that this local optimum could be found between R_ext_ of 0.5 and 2.5 kΩ (with best performing MFCs observed for 1.0 kΩ) and was maintained until 15^th^-16^th^ week of operation. At the same time 50 Ω MFCs, which appeared to show the highest power performance in stage 1, underperformed as compared to MFCs operating under 0.5–2.5 kΩ in the initial period of stage 2 (weeks 9–11), but established a local optimum at the end of the experimental period (weeks 14–17) and outperformed the MFCs matured under higher R_ext_ values.

The overall decreasing trend of power output and simultaneous increase of R_int_ observed in all MFCs in stage 2 resulted from the observed accumulation of inorganic salts on the cathode surfaces (Supporting information – Figure S1), as previously reported for this particular MFC design [28]. Therefore, at the end of stage 2, the cathodes were washed *in situ* with copious amounts of distilled water. As a result, the 50 Ω biofilm was brought back to show the highest power. The power profile observed across all of the MFCs in week 17 was almost identical to the one observed for the 4th week (Fig. 3.4 and 3.17), confirming that divergence of the data during stage 2 was partially a result of the deteriorating cathodes and indicating long-term effect of R_ext_ on biofilm performance. The analysis of the regression surface of power and R_int_ suggests that the deterioration rate of the cathode was increased in the 50 Ω and decreased in the 0.5–2.5 kΩ - matured MFCs. This can be explained by the occurrence of electroosmotic drag, which is related to the power output and could lead to faster accumulation of salts at the cathode observed in the best performing (50 Ω) MFCs [44,45]. In fact, the highest amount of salt deposits at the cathode surface were observed for 50 Ω MFCs, while the lowest for OC control and also 500–5000 Ω MFCs, indicating the interdependence between electroosmotic drag and MFC performance (Figure S1). The cathode performance adds another dynamic factor to the complexity of the microbial fuel cells.

### 3.3 Dynamic electrochemical profile - simultaneous effect of R_ext_ and R_int_ resistance on MFC performance

Data shown in Fig. 4 display a particular type of electrochemical profile – the pattern developed throughout time by all biofilm communities, reflecing dependence of the MFC electrochemical parameters on controlled (external) and developed (internal) resistance. The results show that the highest power efficiency of the MFCs was achieved for the biofilm which developed an internal resistance lower than 300 Ω, and such low R_int_ was only present when the biofilm was matured under R_ext_ between 50 and 1000 Ω. Another local optimum was found for the biofilm matured under R_ext_ between 1000 and 2500 Ω, which developed an R_int_ between 407.2 and 701.8 Ω. Notably, biofilms developed under lower R_ext_ values produced the highest current and the lowest OCV, while the biofilms matured under higher values of R_ext_ showed lower current and higher OCV. These results could be explained by the fact that the biofilm subjected to the effects of the low external resistance (higher anode potential) develops a biofilm with a different composition of electroactive species, as shown by previous studies for both biofilm [[38], [39], [40]] and planktonic communities [38]. It was shown that the strategy of maturing the biofilm under R_ext_ values lower than the lowest observed R_int_ may be beneficial in the long term and this is (to the best of the authors' knowledge) the first report where these phenomena have been demonstrated. Data reported in previous research focused on maturing the biofilm in R_ext_ > R_int_ conditions [22,[24], [25], [26], [27]]. Furthermore, when comparing profiles obtained in two stages of experiment, it can be concluded that the biofilm maintained its electrochemical properties even though the environment was dynamically changing over time. The obtained profiles were similar, including the general decreasing trend in performance, current and OCV, probably due to cathodic salt accumulation, as previously described.

These findings are crucial in defining the appropriate inoculation strategy and protocols for assessing and predicting the MFC performance. The internal resistance proved to be more effective parameter to determine the best performing MFCs than OCV. Nevertheless, reaching high OCV suggests that system may be better balanced and more effective in long term operation, when dynamic changes in cathode performance may occur. The data also shows, that reaching higher potential within MFC environment is not as important as acquiring the biofilm with a desirable structure and community composition as reflected by R_int_, which allows the MFC to reach a higher current production rate and density. This finding suggests that the electrochemical biofilm properties developed over time, are as important as the composition of the electroactive consortia, which also changes dynamically over time [[46], [47], [48]].

### 3.4 Biofilm microarchitecture

The biofilm structure and composition was assessed at the end of experiment (after phase 2 - switching R_ext_ to optimal values) using environmental scanning electron microscopy, as shown on Fig. 5. The most notable changes were in the form of inorganic precipitates. The MFCs matured under lower R_ext_ (50, 500, 1000 Ω) showed no or negligible amounts of such precipitates. Maturing the biofilm under 2.5 kΩ and above resulted in increased amounts of graupel-shaped crystals embedded into the biofilm structure, comprising mainly Na, Cl, Mg, P and Ca, as determined by energy-dispersive X-ray spectroscopy. These salts were present in the highest amounts under open circuit conditions and in all reported cases, adjacent to neighbouring bacterial cells. Such finding may suggest a tendency from the anodic community to accumulate or induce formation of precipitates at very low or no current flow (see Fig. 5). This phenomenon could be one of the factors that contributed to the development of higher R_int_ over time for MFCs that had matured under higher R_ext_, since the crystals are considered to be non-conductive and may have affected the resistivity of the biofilm.

The differences in biofilm microarchitecture were also most notable when comparing high R_ext_ (5 kΩ, 10 kΩ and OC) with low R_ext_ MFCs. Images acquired for high R_ext_ anodic biofilm and OC control, revealed development of looser structures, rich in EPS and with visibly larger water channels in comparison to the low R_ext_ anodic biofilms. In contrast, the lowest R_ext_ (50 Ω) MFC developed a dense and uniform biofilm structure with little EPS content. Zhang et al. [22], quantified the EPS content and reported its inverse relationship with external resistance, which is not in line with the findings of the current study. In that previous study, the results were normalised to the surface area of the carbon cloth electrode (as opposed to the biomass), in addition to using a different R_ext_ range (10–1000 Ω).

In addition to the influence of R_ext_ on the anodic community composition, as previously reported [24,26,27,49], the current study suggests that the biofilm microarchitecture was affected by R_ext_ and played an important role in the evolution of the electrochemical MFC parameters over time, reaching a desirable power efficiency. The observed differences between the lower and higher ranges of R_ext_, as well as the OC control, indicate that beyond a point, the biofilm was irreversibly affected by its initial maturing conditions within the timeframe of experimental period.

### 3.5 Quantitative analysis of dynamic changes in biofilm behaviour

**Fig. 6:**
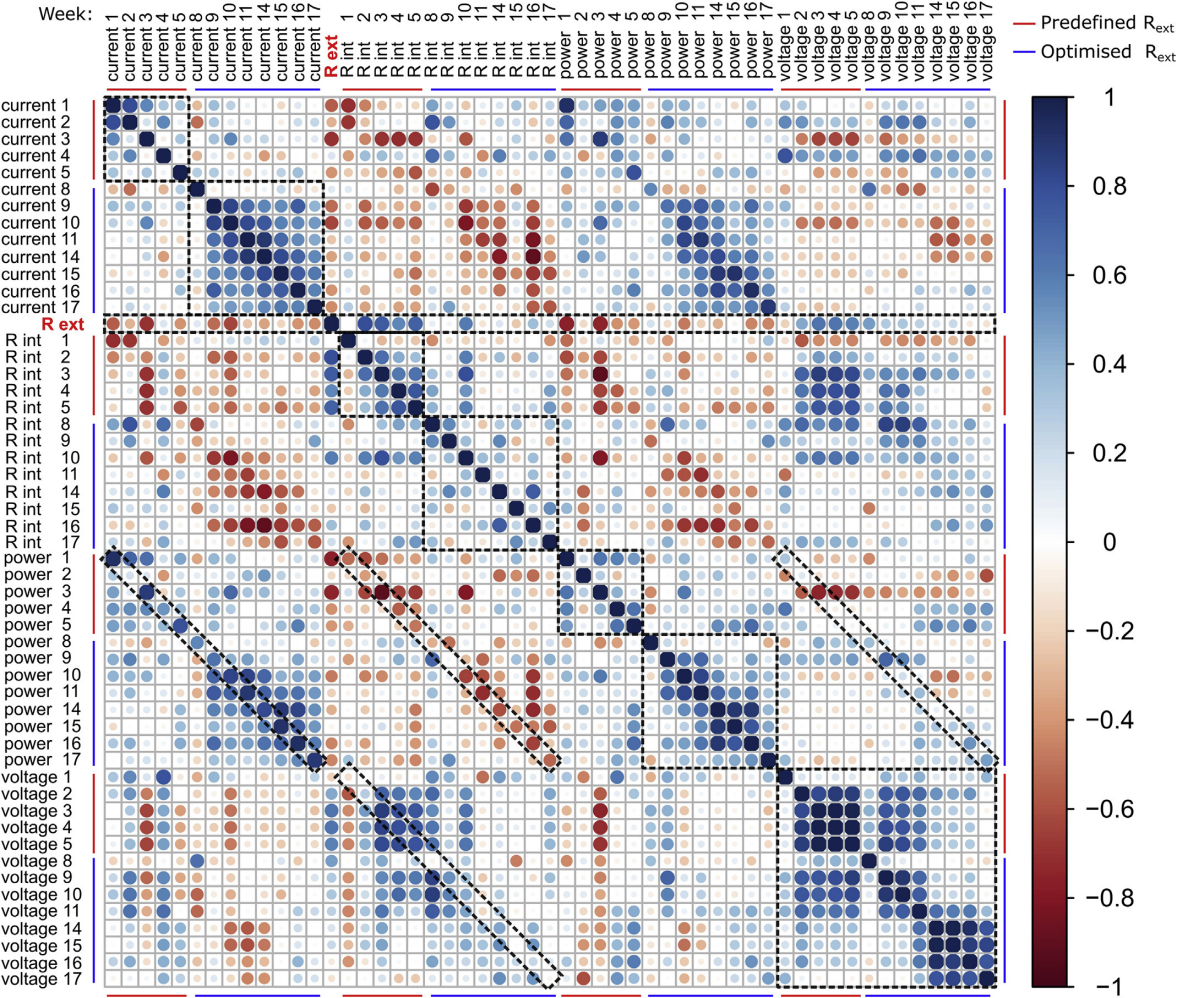
Dynamic changes in correlation of electrochemical parameters reported for the whole population of MFCs in time. The numbers following each label correspond to week of operation, while colour intensity and diameter of the circle corresponds to the Pearson's correlation coefficient (r), as shown on colour key. Inverse correlation is reflected by negative R values, while positive correlation is reflected by positive r values. The following criteria were used to determine the strength of the correlation: weak (±0.1 to ±0.3), moderate (±0.3 to ±0.7), strong (±0.7 to ±0.9), very strong (±0.9 to ±1.0). Dashed lines indicate the regions of interest, which are discussed in the text. (For interpretation of the references to colour in this figure legend, the reader is referred to the Web version of this article.)

To investigate the dynamics of changes recorded for electrochemical parameters of the biofilm, correlation analysis has been conducted, as shown on Fig. 6. In the first stage of biofilm maturing, when fixed R_ext_ values were applied, positive correlation (R = 0.80) was only found for current profiles recorded in the first two weeks, which we consider as a startup period of the MFCs. The correlation coefficient gradually decreased afterwards, confirming that most dynamic changes took place during the first 5 weeks of biofilm maturing. A similar pattern was observed for the power output, while the OCV profiles reached the highest correlation coefficients (0.82–0.99) among all of the parameters, starting from the 2nd week of biofilm maturing. In contrast, following the 2nd week a positive correlation was observed for R_int_ (0.35–0.76) and was decreasing with the increase of time; i.e. two neighbouring R_int_ profiles (variables) were showing the highest correlation. These results suggest, that internal resistance was smoothly changing from one state to another due to the development of the biofilm growth. It can be also concluded, that current was the most dynamic biofilm parameter, while the OCV was the most stable one. Therefore, the data suggest that the power output of the MFCs was rather not affected by the redox potential of bacterial enzymes and resulting electrode overpotentials. This statement is supported by finding a positive correlation between power and current (0.51–0.93, excluding week 2) and negative correlation with R_int_ (−0.38 to −0.91, excluding week 9), while not even a weak correlation was found between OCV and power output. Interestingly, the OCV was very well correlated with the R_int_ during the first weeks of operation reaching R values of 0.77–0.86 following 2 weeks of biofilm maturing. Thus, R_int_ was the main factor determining the potential of the MFCs and reaching high OCV values were not determining for the power performance.

When simulated MPPT method was used to control the power performance of the MFCs in stage 2 the biofilm have readjusted its metabolism to the new conditions. Since during this period the R_ext_ was varying to fit the R_int_, the dynamics of the changes in electrochemical biofilm properties have been partially inhibited. As a result, significant correlation was recorded for current throughout the second stage and reached between 0.40 and 0.89. The highest correlation coefficients were observed for the neighbouring time points and decreased with time, which suggest smooth (less dynamic) evolution of the biofilm from one state to another. In contrast, no significant correlation was observed when the changes of R_int_ were followed in time (stage 2), while significant correlation for this parameter was observed in stage 1. This suggests that when the simulated MPPT procedure was applied, temporal and chaotic changes in R_int_ occurred. Those changes which may have resulted from dynamic biofilm adaptation mechanisms coupled with ongoing changes in cathode performance. However, they were not reflected by the dynamic behaviour reported for current. Although strong negative correlation values were reported for R_int_ and power in that period, power and current were also negatively correlated with original (stage 1) R_ext_ where weak to moderate correlation was reported (R between −0.3 and −0.7). Therefore, as previously shown on Fig. 3, Fig. 4, the external resistance affects the properties and activity of biofilm, which thus becomes irreversibly affected by its preliminary environmental conditions. Nevertheless, the overall correlation between R_ext_ and any other investigated parameter was the strongest in the first stage of operation. The highest (negative and positive) correlation coefficients were reported for 3rd week of operation, which we believe is the most significant period required to develop healthy and well-performing electroactive biofilm in MFC. Although MPPT method proved to be an efficient way of reducing the startup time and improving the performance of the MFCs, its application requires dedicated electronic circuit which affects the cost-efficiency of system [50,51]. In this study, the best performing biofilm was developed when matured under 50 Ω - an R_ext_ approximately 5 times lower than their lowest R_int_, which we believe was a result of outstanding adaptive mechanisms of electroactive bacteria. Therefore, knowledge on internal resistance of the system prior to start-up is crucial for the field-applications, where the cost and available infrastructure may be the limiting factors.

## 4 Conclusions

Maturing the biofilm under various R_ext_ caused several changes in biofilm behaviour. It was shown, that growing the biofilm under higher suboptimal R_ext_ had an adverse effect on its properties and activity. Maturing the biofilm under lower, yet suboptimal R_ext_, resulted in improved R_int_ and power output. The most dynamic changes in electrochemical properties of the biofilm were observed in the first 5 weeks of operation. Implementing MPPT procedure after initial period of maturing was not sufficient to change the electrochemical profile-effect of the MFCs, i.e. dependence of the biofilm activity on initial (stage 1) R_ext_ values. The connected R_ext_ had a significant effect on biofilm three-dimensional structure and composition. These findings are important for developing the appropriate inoculation and maturing strategies to maximise the performance of MFCs.
