# Supplementary material for: Dynamic evolution of anodic biofilm when maturing under different external resistive loads in microbial fuel cells. Electrochemical perspective
Source: J Power Sources. 2018 Oct 1;400:392–401. doi: 10.1016/j.jpowsour.2018.08.031 (PMC6358148; doi:10.1016/j.jpowsour.2018.08.031)
Supplement: Supplementary Data [file mmc1.pdf]

Supporting information

Dynamic evolution of anodic biofilm when maturing under different external resistive loads in microbial fuel cells. Part I – electrochemical Perspective

Grzegorz Pasternak*[*a,b]*, John Greenman*[a]*, Ioannis Ieropoulos*[*a]

[a]Bristol BioEnergy Centre, Bristol Robotics Laboratory, Coldharbour Lane, BS16 1QY Bristol, UK.

[b]Faculty of Chemistry, Wrocław University of Science and Technology, Wyb. Wyspiańskiego 27, 50-370, Wrocław, Poland.

[*]Corresponding authors: grzegorz.pasternak@pwr.edu.pl, ioannis.ieropoulos@brl.ac.uk

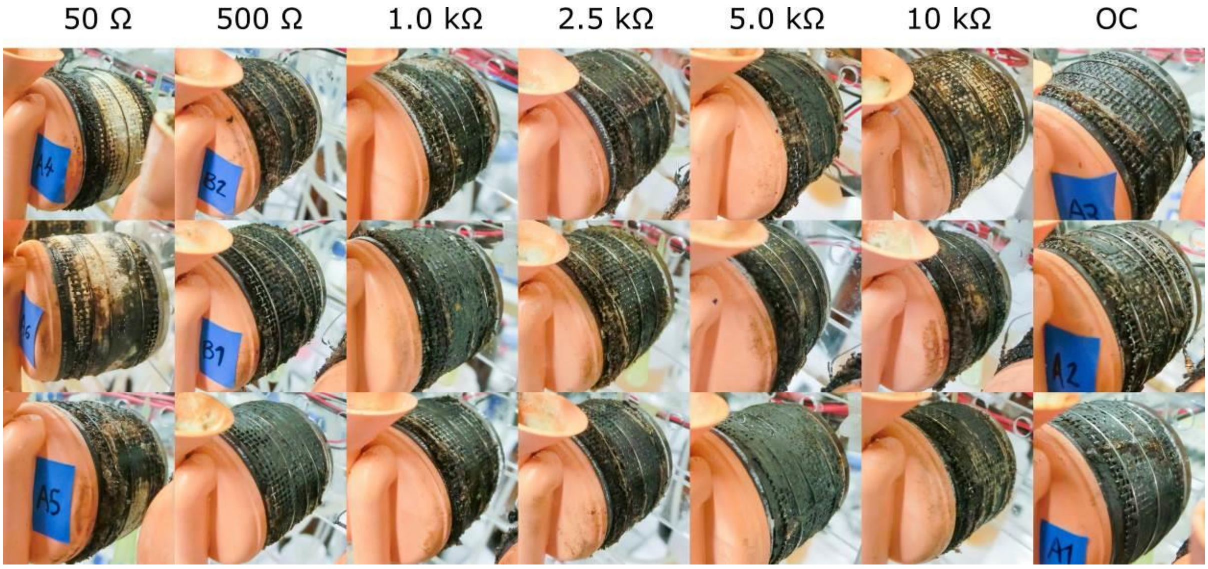

Figure S1. Deteriorated cathodes, indicating the occurrence of electroosmotic drag and dependence of the amount of salt deposits at the cathode surface on R_{ext}.

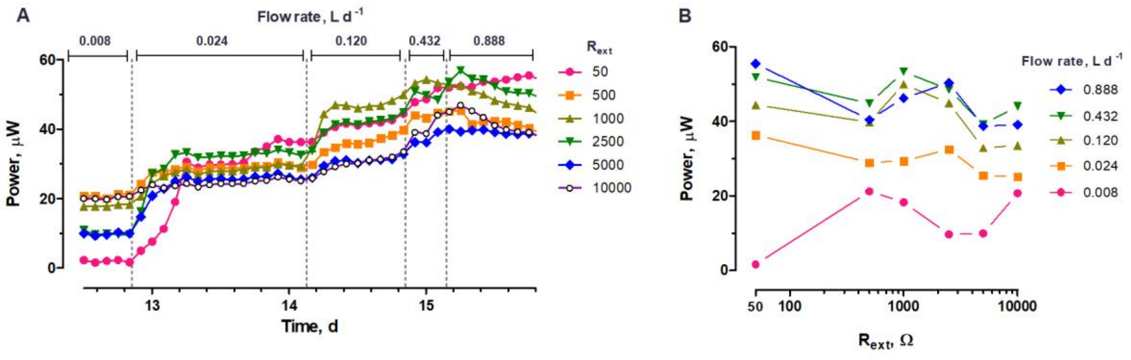

Figure S2. Real time temporal performance of MFCs operated under gradually increasing flow rate (A) and relationship between the R_{ext}, power and flow rate (B). The experiment was completed under conditions where the deterioration of the cathodes was noticed, thus the data represent median values. For reasons of clarity, the range of error bars is not shown.
